# Implementation of Clinical Pharmacy Services in Primary Health Care: A Scoping Review

**DOI:** 10.1111/jep.70285

**Published:** 2025-09-25

**Authors:** João Pedro Vasconcelos Paolinelli, Taiana de Alencar, Kérilin Stancine Santos Rocha, Mariana Linhares Pereira, Genival Araujo dos Santos Júnior

**Affiliations:** ^1^ Graduate Program in Pharmaceutical Services and Policies Federal University of Espirito Santo Alegre‐ES Brazil; ^2^ Laboratory of Innovation for Healthcare (Linc). Postgraduate Program in Pharmaceutical Assistance (PPGASFAR) Federal University of Espírito Santo Vitória ES Brazil; ^3^ Federal University of São João del – Rei Divinópolis MG Brazil

**Keywords:** Implementation Science, Pharmaceutical Care, Pharmaceutical Services, Primary Health Care

## Abstract

**Rationale:**

The implementation of Clinical Pharmacy Services in healthcare systems is multifactorial and complex. Some studies summarize the implementation research of these services in both outpatient and inpatient contexts. However, the literature lacks a comprehensive synthesis to understand how the implementation process occurs in Primary Health Care.

**Aim:**

To map, at an international level, the studies on the implementation of Clinical Pharmacy Services in Primary Health Care.

**Method:**

This scoping review followed the recommendations of the JBI Manual for Evidence Synthesis for Scoping Reviews. A systematic search was conducted in May 2023 across six electronic databases, grey literature, and the references of the selected studies. Original studies that described the implementation process of Clinical Pharmacy Services in Primary Health Care were eligible for inclusion. Two researchers independently assessed the studies based on eligibility criteria and extracted data from the selected articles. Data were presented descriptively.

**Results:**

A total of 97 studies were included, with 85.6% (*n* = 83) conducted in developed countries. A significant emphasis was observed on qualitative and mixed‐methods research, accounting for 46.4% (*n* = 45) of the studies. Only 22.7% (*n* = 22) of the studies utilized theoretical models as research guides, while 96.9% (*n* = 94), did not report on the implementation phase. Comprehensive Medication Management (36.8%, *n* = 35) was the most cited type of service. The most assessed implementation outcome was feasibility, at 73.2% (*n* = 71). A total of 47.4% (*n* = 46) assessed outcomes related to the implemented service, which included clinical, economic, and/or humanistic aspects.

**Conclusion:**

This study revealed numerous publications on the implementation of clinical services provided by pharmacists in primary care. These findings can guide future research on the implementation of these services and highlight the need for developing and underdeveloped countries to explore this topic further.

## 1 Introduction

The role of pharmacists in Primary Health Care (PHC) has evolved beyond managerial activities [1]. There is a notable increase in the clinical involvement of these professionals in various settings, integrated into multidisciplinary teams, by providing different Clinical Pharmacy Services (CPS) [2]. These services aim to promote, protect, and recover health, prevent diseases, and resolve issues related to pharmacotherapy through the assessment of the indication, effectiveness, safety, and adherence to medicine use [3, 4].

Studies report the provision of CPS for various populations, health conditions, and different levels of care, indicating positive clinical, economic, and humanistic benefits [5, 6, 7, 8, 9, 10, 11, 12, 13]. In PHC, the provision of CPS enables disease screening, treatment of self‐limiting health issues, and contributes to the multidisciplinary team approach, particularly in the management of patients with chronic health conditions, reducing the burden on physicians and preventing unnecessary hospitalizations and costs for patients and health systems [14, 15, 16, 17, 18].

However, such services are not uniformly present in health systems around the world, and when they are, they often do not complete their implementation process or do not sustain themselves in actual clinical practice [19, 20]. The large‐scale expansion of these services in PHC is hindered by multiple contextual factors related to limited resources and the poor infrastructure of health systems, lack of time for professionals, low levels of clinical competencies, inadequate access to patient data, among others [21, 22, 23, 24]. In summary, there is a gap between the results of scientific research conducted in controlled environments and their translation/incorporation into clinical practice [24, 25, 26].

This gap can be addressed by implementation science, an area of knowledge that deals with the systematization of research results and other evidence‐based practices to improve the quality and efficiency of health services and care [28, 29, 30]. Therefore, research on this topic focuses on the patient, the care provider, organizations and public policies, considering different realities, particularities and factors that are part of the extensive scenario of an actual clinical practice, thus configuring itself as a facilitator for the translation of the evidence generated by controlled studies, to the real scenarios of actual practices [20, 29, 31].

To better understand the implementation process of the CPS, other systematic reviews summarized and evaluated the original implementation studies in outpatient [32] and inpatient settings [32, 33, 34]. In PHC, Brandt et al. [35] also mapped the literature in search of these studies, but focused on Medication Review services in community pharmacies. As such, the literature currently lacks a comprehensive mapping of studies on the implementation of CPS within the context of PHC. Therefore, this study aims to address this gap by systematically mapping the relevant studies at an international level. This study innovates in relation to current publications by expanding the search scope to the various types of CPSs and places of service provision in PHC.

## 2 Method

This is a scoping review conducted in accordance with the recommendations of the JBI Manual for Evidence Synthesis for Scoping Reviews [36]. The review was reported following the Preferred Reporting Items for Systematic reviews and Meta‐Analyses extension for Scoping Reviews ‐ (PRISMA‐ScR) criteria (Supporting Information 1) [37]. The review protocol was registered in the Open Science Framework (registration number: doi:10.17605/OSF.IO/N25EA) [38].

A preliminary search was conducted in PubMed/MEDLINE, as well as in the Cochrane Database of Systematic Reviews and the JBI Evidence Synthesis to find similar studies. We found only one scope review protocol on this topic, but with its scope restricted to studies that evaluate barriers and facilitators of the implementation of CPS in PHC [39]. Our mapping is based on a broader scope than the aforementioned, since there was no restriction on the different objectives, methods, and results evaluated in our implementation science‐based research [40, 41].

### 2.1 Research Question

The acronym Population‐Concept‐Context (PCC) was used for the elaboration of the research question [36].

- *Population*: licensed pharmacists working with the provision of CPS at the PHC level.
- *Concept*: refers to the CPS (services provided by the pharmacist alone, or those in which the pharmacist effectively participates in the multidisciplinary team). The following CPS were considered: Medication Review; Comprehensive Medication Management; Medication Reconciliation; Drug Dispensing; Management of Minor Illness; Health Education; Drug Monitoring; Disease Screening; Disease Management [1, 3, 4, 42, 43, 44].
- *Context*: refers to PHC. This is understood as the first point of contact, offering comprehensive care, characterized by the promotion and protection of health, the prevention of diseases, diagnosis, treatment, rehabilitation, harm reduction, and health maintenance, with the aim of developing comprehensive care that positively impacts the health of individuals and collectives. The offer of this set of actions should be as close as possible to the daily environment of individuals, families and community [45, 46]. It is important to highlight that the universal concept of PHC comes up against variations in structure, policy, organization, financing and practices carried out in different countries. Among these differences, we highlight the non‐separation of outpatient care into levels (between that provided by nonspecialist professionals and that provided by specialists), as well as the type of first‐contact health unit, which can be a community pharmacy, outpatient clinic, autonomous clinics, other clinics, and health centers of non‐specialized care [47, 48, 49, 50, 51, 52, 53].

Thus, this scoping review was guided by the following question: “What has been published about the implementation of clinical pharmacy services in the context of primary health care?”

### 2.2 Search Strategy

A systematic search was carried out in May 2023, in the following electronic databases: EMBASE, MEDLINE (PubMed), Cochrane Central Register of Controlled Trials (CENTRAL), Web of Science, Scopus, and Latin American and Caribbean Health Sciences (LILACS). The grey literature was also consulted via the “The Digital Library of Theses and Dissertations of the University of São Paulo”. In addition, the references of the included studies were analyzed manually in search of additional studies.

The search strategy was set up and aligned with the research objective and question, resulting in four groups of terms that encompass: “Implementation Science”, “Clinical Pharmacy Services”, “Pharmacists”, and “Primary Health Care”. The Medical Subject Headings (MeSH) and the Embase Subject Headings (Entree) were used to define the descriptors, their synonyms, and other keywords cited by our references [1, 3, 4, 27, 28, 29, 30, 31, 42, 43, 44, 45, 46, 47, 48, 49, 50, 51, 52, 53, 54]. The entire search strategy was adapted to the operation‐specificities of each database (Supporting Information 2).

### 2.3 Eligibility Criteria

#### 2.3.1 Inclusion Criteria

Original, observational, and experimental studies, either qualitative or mixed‐methods, with no restrictions on publication date or language, that addressed the implementation process of CPS in PHC.

#### 2.3.2 Exclusion Criteria

The following were excluded: literature reviews, study protocols, non‐original articles such as letters, editorials, congress abstracts, and posters; studies that only assessed the impact of pharmacists on clinical, economic, and/or humanistic outcomes without addressing aspects related to implementation; studies with unavailability of full texts; studies conducted simultaneously in PHC and other levels of care where PHC results could not be separately collected and analyzed; studies conducted in outpatient clinics, specialized clinics, and offices that were not restricted to the primary care level.

### 2.4 Study Selection

All authors underwent training to improve reliability among the reviewers of this review. After searching the databases, all identified references were grouped and exported to Rayyan, an online tool that optimizes the initial selection process of references by using a semi‐automation process [55]. With the help of this tool, the steps of removing duplicates and sorting by titles and abstracts were conducted. Two researchers independently screened titles and abstracts based on the eligibility criteria. Then, the full texts of the previously selected articles were independently read and evaluated by the two researchers. A third researcher monitored the selection stages to resolve any disagreements between the first two. The PRISMA‐ScR flow diagram [37] was used to report the search results and record the reasons for excluding articles.

### 2.5 Data Extraction

Data were extracted using a form previously designed and tested by the authors. The testing phase involved data extraction from ten studies by two independent researchers for calibration purposes. After this stage, all selected articles were independently analyzed by two researchers, and in case of disagreement, a third researcher was consulted. The variables collected included: author(s), country, objective(s), study design, participants, type of CPS, use of models as implementation guides, implementation phase, location of CPS delivery, and implementation outcomes.

The “implementation phase” variable was interpreted by the authors of this review using the definition proposed by Handley, Gorukanti, and Cattamanchi [56], if the study did not explicitly describe it. These authors propose three main phases to guide, plan, and conduct implementation research: Phase 1 – Pre‐implementation planning: focuses on engaging stakeholders in the process and planning the translation of evidence; Phase 2 – Designing the implementation strategy: involves using theories/conceptual models to identify obstacles and facilitators to implementation, adjust the process and organize the description of outcomes; Phase 3 – Evaluating the implementation strategy: assesses the outcomes of the implementation process, determines and explains whether the strategies were effective.

For the variable “implementation outcomes,” the conceptual taxonomy by Proctor et al. [40] was used to identify the different types of implementation process outcomes, such as acceptability, adoption, appropriateness, feasibility, penetration, fidelity, cost‐implementation, and sustainability. In addition, the theoretical framework of Kozma, Reeder and Schulz [41] was used to identify the outcomes of the service, classified into clinical, humanistic, and economic.

### 2.6 Data Analysis

A comprehensive descriptive synthesis of the results was presented in table form to facilitate visualization and understanding. Descriptive statistics, using absolute and relative frequencies, were employed for reporting and interpreting the variables extracted from the selected studies. Consistent with JBI and PRISMA‐ScR recommendations, no individual risk of bias analysis was performed on the selected studies.

## 3 Results

### 3.1 Study Selection

A total of 3,147 studies were retrieved from electronic databases and the gray literature, of which 97 studies were included in this review. The details of the search, identification, and selection process of the studies are described in Figure 1, available in Supporting Information 3.

### 3.2 Characteristics of the Selected Studies

A total of 85.6% of the studies (*n* = 83) were conducted in developed countries, with the United States of America, Australia, and the United Kingdom standing out, respectively accounting for 32% (*n* = 31), 14.4% (*n* = 14), and 11.3% (*n* = 11) of the total publications. Among the studies conducted in developing countries, Brazil stands out with 10.3% (*n* = 10) [57].

Regarding the objectives of the studies, 47.4% (*n* = 46) investigated barriers and facilitators that determine the implementation process, as well as the perceptions, attitudes, opinions, perspectives, and experiences of various stakeholders involved (patients, health managers, pharmacists, general practitioners, nurses, assistants, among others) in the implementation process of CPSs. The remainder was divided among studies focused on the description, analysis, and evaluation of strategies to be implemented or already implemented.

The most commonly used study designs were qualitative and mixed methods, which together represent 46.4% (*n* = 45) of the selected studies. Among the exclusively quantitative studies, 19.6% (*n* = 19) were observational, and 13.4% (*n* = 13) were interventional [58]. The remaining studies, 20.7% (*n* = 20), did not describe their design.

Only 22.7% (*n* = 22) of the studies used at least one theoretical reference, model, or framework at some stage of the research process to guide implementation or to categorize and explain the results.

The main CPSs reported in the selected studies were Comprehensive Medication Management and Medication Review, identified in 36.8% (*n* = 35) and 27.8% (*n* = 27) of the studies, respectively. Other CPSs mentioned included health screening in 10.3% (*n* = 10), Health Education in 9.3% (*n* = 9), Medication Reconciliation in 7.2% (*n* = 7), Disease Management in 6.2% (*n* = 6), Management of Minor Ilness in 3.9% (*n* = 3), Drug Monitoring in 3.09% (*n* = 3), and Drug Dispensing in 2.06% (*n* = 2). Additionally, 13.4% (*n* = 13) of the studies reported more than one CPS. In contrast, 8.2% (*n* = 8) did not specify the type of CPS, referring to them using general terms.

The most common locations for service delivery were community pharmacies (48.5%, *n* = 47) and primary health care units (clinics, offices, and primary care outpatient facilities, as well as basic health units) (44.3%, *n* = 43). Other studies reported the provision of CPS in households (6.2%, *n* = 6) and barbershops (1.03%, *n* = 1). One study did not report this information.

A total of 94 studies (96.9%) did not report the implementation phase of the CPS. Through the theoretical framework used by the authors [56], we observed that 56.4% (*n* = 53) of the studies reported a single implementation phase. Of these, (5.3%, *n* = 5) provided information on Phase 1, which represents the pre‐implementation stage; 16% (*n* = 15) of the studies reported Phase 2, in which the implementation strategy is projected; and 35.1% (*n* = 64) of the studies reported Phase 3, in which the results of the implementation strategies are evaluated. Additionally, it is noteworthy that the remaining studies (43.6%, *n* = 41) reported, concurrently in the same article, two or three phases of the implementation process.

All studies evaluated at least one implementation outcome proposed by Proctor et al. [40] with an emphasis on the evaluation of feasibility (73.2%, *n* = 71) and acceptability (35.05%, *n* = 34). Furthermore, it is highlighted that the outcomes of appropriateness (11,3% *n* = 11), adoption (10.3%, *n* = 10), fidelity (8.2%, *n* = 8), penetration (7.2%, *n* = 7), cost‐implementation (7.2%, *n* = 7), and sustainability (9.2%, *n* = 9) were exclusively evaluated by studies conducted in developed countries. Regarding the service outcomes proposed by Kozma, Reeder, and Schulz [41], 47.4% (*n* = 46) reported clinical, economic, and/or humanistic outcomes.

The characteristics of the selected studies are detailed in Table 1, available in Supporting Information 4.

## 4 Discussion

This scoping review broadly exposed the current global landscape of implementation research on clinical pharmacy services in primary health care. Most of this study occurred in developed countries, indicating that the implementation of clinical pharmacy services in primary health care settings is still progressing slowly in developing and underdeveloped countries, or that these services are not being published in the scientific article format. This fact is possibly related to the lower economic indicators of these countries, which impact the pharmaceutical workforce, patient access to medicines, specialized training in the clinical area, human resources, infrastructure, and investment in research [156]. Thus, in addition to the need for more implementation studies in these countries, it is emphasized that the reporting, publication, and scientific dissemination of this type of study should be promoted.

Regarding the study designs of the implementation research, there is a predominance of qualitative and mixed‐method studies, which is corroborated by Brand et al. [35]. These are important for analyzing variables that are not effectively measurable by exclusively quantitative studies, such as the determinants of the implementation process, the assessment of opinions, experiences, perspectives, and acceptability of stakeholders, as well as the feasibility of the implemented intervention [157]. Additionally, it is noteworthy that this finding aligns with the number of studies aimed at analyzing the aforementioned variables. The points that qualitative studies address are already well‐studied in the implementation of CPS in various practice scenarios [157]. Thus, there is a need to advance knowledge of implementation from other perspectives, such as effectiveness, costs, and sustainability evaluation.

The present review pointed out that most of the selected studies did not report on the use of models/theories or frameworks at any stage of the research process. Pereira et al. [32] corroborate this result by exposing in their systematic review that 75% of the implementation research of CPS in outpatient and hospital contexts do not report the use of these models. The use of a theoretical model in implementation research serves as a roadmap for planning and executing implementation strategies, increasing the efficacy/efficiency of research, generalization, and interpretation of results [158]. This finding should be a topic of discussion among researchers in the field to establish a recommendation for the use of these models in CPS implementation research, as relying on empirical guidance can reduce the chances of success [158].

Similar to the aforementioned data, few studies reported the implementation phase of the intervention. In line with this finding, other systematic reviews also identified this deficiency in CPS implementation studies in outpatient and inpatient contexts [32, 33]. Through the theoretical framework used, we observed that most studies describe only one implementation phase in their articles. This fact may be attributed to the complexity of implementation research, which may sometimes require cut‐offs for in‐depth data analysis and to meet scientific publication standards [20]. The implementation phases proposed by Handley, Gorukanti, and Cattamanchi [56] offer a methodology that assists field researchers in outlining and conducting scientific investigations on implementation. The three proposed phases are not static; they can occur simultaneously [56]. This characteristic explains part of the studies selected here, which reported, in the authors’ interpretation of this study, two or three simultaneous phases in their articles.

The results regarding the use of theoretical models and the reporting of implementation phases suggest that publications on the implementation of CPSs at different levels of care lack important information that directly impacts the interpretation of results and the replication or adaptation of the generated evidence [159]. This finding aligns with the systematic review by Pinnock et al. [160] that exposed the inadequacy of context description, absence of theoretical models, and insufficient information on how the intervention was promoted in asthma self‐management implementation studies. Thus, it is suggested that researchers use tools that assist in reporting implementation studies in the area, such as the Standards for Reporting Implementation Studies (StaRI), and that scientific journals consider requiring the submission of a checklist like the one mentioned above to fill this gap and reduce hurdles in disseminating evidence to the scientific community [159, 161].

Most studies on the implementation of CPS in PHC utilized the community pharmacy setting as the location for service delivery. This finding is consistent with government initiatives in developed countries, such as the USA, Australia, and the UK, to integrate community pharmacies into their healthcare systems [50, 51, 162]. Through political agreements between public and private entities, these countries invest in infrastructure, human resources training, remuneration for CPS provision, among other strategies, to ensure that these locations are among the first points of entry into the healthcare system and contribute to the screening and monitoring of diseases in partnership with general practitioners [162]. Therefore, it is expected that this strategy used by developed countries will be the subject of discussions, studies, research, and policy‐making that regulate and decentralize CPS to community pharmacies in developing countries, so as to enhance their implementation in PHC.

This review revealed that nine different CPSs were found in PHC, mainly Comprehensive Medication Management and Medication Review. This can be explained by the fact that these two CPSs are well‐studied and have a robust body of scientific evidence. The literature has demonstrated the positive impact of these services on clinical, economic, and humanistic outcomes for various health conditions [6, 7, 8, 9, 10, 13]. Moreover, these two CPSs may be particularly suitable for addressing conditions sensitive to PHC, especially in monitoring chronic health conditions, benefiting individuals, professionals, and the healthcare system [7, 8, 9, 10, 43, 44, 45, 46, 47].

A large portion of the studies focused on evaluating the feasibility and acceptability of implementing the services. These measures are important for analyzing the research conduct, especially in the early and intermediate phases of the process, but they are not sufficient to ensure the success of implementation [40]. Measures such as fidelity (whether the implementation protocol was correctly followed), penetration (capacity to serve the population), and cost‐implementation (financial impact) directly influence sustainability (the likelihood that the service will be maintained in the future) [40]. The studies that attempted to evaluate all implementation outcomes were few, and exclusively conducted in developed countries. These data indicate the mismatch in the pace of CPS implementation in PHC between developed, developing, and underdeveloped countries.

Additionally, it is noteworthy that, regarding service outcomes, almost half of the studies evaluated clinical, economic, and/or humanistic outcomes. In general, there is a relationship between the implementation outcomes proposed by Proctor et al. [40] and the service outcomes proposed by Kozma, Reed, and Shulz [20, 41]. Thus, clinical, economic, and humanistic outcomes can be used as dependent variables for assessing process indicators and implementation success, and therefore, are important to be evaluated by implementation studies [20].

The authors acknowledge that this scoping review is not without limitations. According to the theoretical framework adopted, this study did not consider some types of services referenced in the literature, such as pharmaceutical prescribing, vaccination, stewardship programs, and services aimed at providing integrative and complementary practices. Moreover, only one database of gray literature was used, and it is unclear how this may have influenced the results.

However, to the best of our knowledge, this is the first review that mapped the literature in search of studies on the implementation of various CPSs in different contexts and realities of PHC. It is emphasized that the use of the conceptual framework for defining CPS allowed the development of a search strategy aligned with the breadth of the research question. Thus, the use of a broad and systematic search engine, followed by rigorous steps for screening, selection, and data extraction, allowed for capturing a vast existing literature capable of answering the research question of the scoping review.

## 5 Conclusions

The extensive mapping conducted by this scoping review has generally exposed what has been published about the implementation of CPS in PHC. Most of the research occurred in developed countries, indicating that the implementation of CPS in the PHC of developing and underdeveloped countries is still progressing slowly, or that these services are not being published in the scientific article format. Implementation research in the area should define more robust methodological criteria, such as the use of conceptual models, to increase the chances of success at all stages of the implementation process and the capacity for replicating/adapting the evidence generated. Moreover, there is a need for these studies to redirect their evaluation focus towards those that seek to assess coverage, cost‐implementation, as well as clinical and humanistic outcomes, to achieve the sustainability of the service in the future. It is expected that the findings of this study will support future research and implementation of CPS, aiming to promote, refine, standardize, and balance the implementation of these services across the different contexts and realities of PHC globally.

## Conflicts of Interest

The authors declare no conflict of interest.

## Supporting information

Supplementary Material 2.

Supplementary Material 3.

Supplementary Material 4_1.
Supplementary Material 4_2.

Supplementary Material 5.

## Data Availability

The data that supports the findings of this study are available in the supporting material of this article.
