# Supplementary material for: Implementation of Clinical Pharmacy Services in Primary Health Care: A Scoping Review
Source: J Eval Clin Pract. 2025 Sep 25;31(6):e70285. doi: 10.1111/jep.70285 (PMC12462563; doi:10.1111/jep.70285)
Supplement: Supplementary file 1 — Supplementary Material 2. [file JEP-31-0-s002.docx]

**Supplementary material 2 –** Search strategy for each database

| **Pubmed** |
| --- |
| ("Implementation Science"[MeSH Terms] OR "Implement"[All Fields] OR "Implementation"[All Fields] OR "Implementation framework"[All Fields] OR "Implementation determinants"[All Fields] OR "Implementation outcomes"[All Fields] OR "Implementation evaluation"[All Fields] OR "Active Implementation Frameworks"[All Fields] OR "Adoption"[All Fields] OR "Quality improvement"[All Fields] OR "Dissemination"[All Fields] OR "complex intervention"[All Fields] OR "Implementation Research"[All Fields] OR "knowledge translation"[All Fields] OR "knowledge transfer"[All Fields] OR "knowledge mobilization"[All Fields] OR "Research utilization"[All Fields] OR "Knowledge Uptake"[All Fields] OR "knowledge Exchange"[All Fields] OR "Knowledge Translations"[All Fields] OR "translation knowledge"[All Fields] OR "translations knowledge"[All Fields] OR "diffusion innovation"[All Fields] OR "diffusion of innovation"[All Fields] OR "dissemination research"[All Fields] OR "Implementation Research"[All Fields] OR "health systems research"[All Fields] OR "operational research"[All Fields] OR "quality improvement research"[All Fields] OR "Translational Research"[All Fields] OR "Knowledge synthesis"[All Fields]) AND ("Pharmaceutical Services"[MeSH Terms] OR "services, pharmacy"[All Fields] OR "Pharmaceutic Services"[All Fields] OR "Pharmaceutic Service"[All Fields] OR "services, pharmaceutical"[All Fields] OR "Pharmaceutical Service"[All Fields] OR "service, pharmaceutical"[All Fields] OR "Pharmacy Services"[All Fields] OR "Pharmacy Service"[All Fields] OR "service, pharmacy"[All Fields] OR "Pharmaceutical Care"[All Fields] OR "care, pharmaceutical"[All Fields] OR "management medication therapy"[All Fields] OR "Medication Therapy Management"[MeSH Terms] OR "therapy management medication"[All Fields] OR "Drug Therapy Management"[All Fields] OR "management, drug therapy"[All Fields] OR "Medication Review"[MeSH Terms] OR "Medication Reviews"[All Fields] OR "review, medication"[All Fields] OR "reviews, medication"[All Fields] OR "Drug Utilization Review"[MeSH Terms] OR "drug-use review"[All Fields] OR "drug use review"[All Fields] OR "Drug-Use Reviews"[All Fields] OR "reviews, drug use"[All Fields] OR "review, drug utilization"[All Fields] OR "Drug Utilization Reviews"[All Fields] OR "reviews, drug utilization"[All Fields] OR "utilization review, drug"[All Fields] OR "Drug Utilization Evaluation"[All Fields] OR "Drug Utilization Evaluations"[All Fields] OR "review, drug-use"[All Fields] OR "review, drug use"[All Fields] OR "pharmacy service, clinical"[All Fields] OR "service, clinical pharmacy"[All Fields] OR "Clinical Pharmacy Services"[All Fields] OR "pharmacy services, clinical"[All Fields] OR "services, clinical pharmacy"[All Fields] OR "Clinical Pharmacy Service"[All Fields] OR "dispensing"[All Fields] OR "drug dispensing"[All Fields] OR "health education"[All Fields] OR "responsible self medication"[All Fields] OR "comprehensive medication management"[All Fields] OR "therapeutic drug monitoring"[All Fields] OR "drug monitoring"[All Fields] OR "medication reconciliation"[All Fields] OR "medicines use review"[All Fields] OR "brown bag review"[All Fields] OR "drug regimen review"[All Fields] OR "home medicines review"[All Fields] OR "medication therapy review"[All Fields] OR "medication management review"[All Fields] OR "disease management"[All Fields] OR "pharmacotherapeutic follow up"[All Fields] OR "medicines management"[All Fields] OR "Cognitive service"[All Fields] OR "Cognitive services"[All Fields] OR "Cognitive pharmaceutical services"[All Fields] OR "Cognitive pharmaceutical service"[All Fields] OR "Community Pharmacy Services"[MeSH Terms] OR "pharmacy, services community"[All Fields] OR "Community Pharmacy, Service"[All Fields] OR "services, community pharmacy"[All Fields] OR "Community Pharmaceutic Services"[All Fields] OR "Community Pharmaceutical Services"[All Fields] OR "Community Pharmaceutical Service"[All Fields] OR "service, community pharmacy"[All Fields]) AND ("Primary Health Care"[MeSH Terms] OR "care, primary health"[All Fields] OR "health, care primary"[All Fields] OR "Primary Healthcare"[All Fields] OR "healthcare, primary"[All Fields] OR "Primary Care"[All Fields] OR "care, primary"[All Fields] OR "Basic health care"[All Fields]) AND ("Pharmacists"[MeSH Terms] OR "Pharmacist"[All Fields] OR "Clinical Pharmacists"[All Fields] OR "Clinical Pharmacist"[All Fields] OR "pharmacist, clinical"[All Fields] OR "pharmacists, clinical"[All Fields] OR "Community Pharmacists"[All Fields] OR "Community Pharmacist"[All Fields] OR "pharmacist, community"[All Fields] OR "pharmacists, community"[All Fields] OR "Retail Pharmacists"[All Fields] OR "pharmacists, retail"[All Fields] OR "Retail Pharmacist"[All Fields]) |
| **Embase** |
| ('pharmaceutical services' OR 'services, pharmacy' OR 'pharmaceutic services' OR 'pharmaceutic service' OR 'services, pharmaceutical' OR 'pharmaceutical service' OR 'service, pharmaceutical' OR 'pharmacy services' OR 'pharmacy service' OR 'service, pharmacy' OR 'pharmaceutical care'/exp OR 'pharmaceutical care' OR 'care, pharmaceutical' OR 'management medication therapy' OR 'medication therapy management'/exp OR 'medication therapy management' OR 'therapy management medication' OR 'drug therapy management' OR 'management, drug therapy' OR 'medication review' OR 'medication reviews' OR 'review, medication' OR 'reviews, medication' OR 'drug utilization review'/exp OR 'drug utilization review' OR 'drug-use review' OR 'drug use review' OR 'drug-use reviews' OR 'reviews, drug use' OR 'review, drug utilization' OR 'drug utilization reviews' OR 'reviews, drug utilization' OR 'utilization review, drug' OR 'drug utilization evaluation' OR 'drug utilization evaluations' OR 'review, drug-use' OR 'review, drug use' OR 'pharmacy service, clinical' OR 'service, clinical pharmacy' OR 'clinical pharmacy services' OR 'pharmacy services, clinical' OR 'services, clinical pharmacy' OR 'clinical pharmacy service' OR 'dispensing' OR 'drug dispensing' OR 'health education'/exp OR 'health education' OR 'responsible self medication' OR 'comprehensive medication management' OR 'therapeutic drug monitoring' OR 'drug monitoring'/exp OR 'drug monitoring' OR 'medication reconciliation' OR 'medicines use review' OR 'brown bag review' OR 'drug regimen review' OR 'home medicines review'/exp OR 'home medicines review' OR 'medication therapy review' OR 'medication management review' OR 'disease management'/exp OR 'disease management' OR 'pharmacotherapeutic follow up' OR 'medicines management' OR 'cognitive service' OR 'cognitive services' OR 'cognitive pharmaceutical services' OR 'cognitive pharmaceutical service' OR 'community pharmacy services' OR 'pharmacy services, community' OR 'community pharmacy, service' OR 'services, community pharmacy' OR 'community pharmaceutic services' OR 'community pharmaceutical services' OR 'community pharmaceutical service' OR 'service, community pharmacy') AND ('implementation science'/exp OR 'implementation science' OR 'implement' OR 'implementation' OR 'implementation framework' OR 'implementation determinants' OR 'implementation outcomes' OR 'implementation evaluation' OR 'active implementation frameworks' OR 'adoption'/exp OR 'adoption' OR 'quality improvement' OR 'dissemination' OR 'complex intervention' OR 'implementation research' OR 'knowledge translation' OR 'knowledge transfer' OR 'knowledge mobilization' OR 'research utilization' OR 'knowledge uptake' OR 'knowledge exchange' OR 'knowledge translations' OR 'translation knowledge' OR 'translations knowledge' OR 'diffusion innovation' OR 'diffusion of innovation'/exp OR 'diffusion of innovation' OR 'dissemination research' OR 'health systems research' OR 'operational research' OR 'quality improvement research' OR 'translational research'/exp OR 'translational research' OR 'knowledge synthesis') AND ('primary health care'/exp OR 'primary health care' OR 'care, primary health' OR 'health care, primary' OR 'primary healthcare' OR 'healthcare, primary' OR 'primary care' OR 'care, primary' OR 'basic health care') AND ('pharmacists' OR 'pharmacist'/exp OR 'pharmacist' OR 'clinical pharmacists' OR 'clinical pharmacist'/exp OR 'clinical pharmacist' OR 'pharmacist, clinical' OR 'pharmacists, clinical' OR 'community pharmacist'/exp OR 'community pharmacists' OR 'community pharmacist' OR 'pharmacist, community' OR 'pharmacists, community' OR 'retail pharmacists' OR 'pharmacists, retail' OR 'retail pharmacist' OR 'pharmacist, retail') AND [embase]/lim |
| **Scopus** |
| ( TITLE-ABS-KEY ( "Implementation Science"  OR  "Implement"  OR  "Implementation"  OR  "Implementation framework"  OR  "Implementation determinants"  OR  "Implementation outcomes"  OR  "Implementation evaluation"  OR  "Active Implementation Frameworks"  OR  "Adoption"  OR  "Quality improvement"  OR  "Dissemination"  OR  "complex intervention"  OR  "Implementation Research"  OR  "knowledge translation"  OR  "knowledge transfer"  OR  "knowledge mobilization"  OR  "Research utilization"  OR  "Knowledge Uptake"  OR  "knowledge Exchange"  OR  "Knowledge Translations"  OR  "translation knowledge"  OR  "translations knowledge"  OR  "diffusion innovation"  OR  "diffusion of innovation"  OR  "dissemination research"  OR  "health systems Research"  OR  "operational research"  OR  "quality improvement Research"  OR  "Translational Research"  OR  "Knowledge synthesis" ) )  AND  ( TITLE-ABS-KEY ( "Pharmaceutical Services"  OR  "services, pharmacy"  OR  "Pharmaceutic Services"  OR  "Pharmaceutic Service"  OR  "services, pharmaceutical"  OR  "Pharmaceutical Service"  OR  "service, pharmaceutical"  OR  "Pharmacy Services"  OR  "Pharmacy Service"  OR  "service, pharmacy"  OR  "Pharmaceutical Care"  OR  "care, pharmaceutical"  OR  "management, medication therapy"  OR  "Medication Therapy Management"  OR  "therapy management medication"  OR  "Drug Therapy Management"  OR  "management, drug therapy"  OR  "Medication Review"  OR  "Medication Reviews"  OR  "review, medication"  OR  "reviews, medication"  OR  "Drug Utilization Review"  OR  "drug-use review"  OR  "drug use review"  OR  "Drug-Use Reviews"  OR  "reviews, drug-use"  OR  "review, drug utilization"  OR  "Drug Utilization Reviews"  OR  "reviews, drug utilization"  OR  "utilization review, drug"  OR  "Drug Utilization Evaluation"  OR  "Drug Utilization Evaluations"  OR  "review, drug-use"  OR  "review, drug use"  OR  "pharmacy service, clinical"  OR  "service, clinical pharmacy"  OR  "Clinical Pharmacy Services"  OR  "pharmacy services, clinical"  OR  "services, clinical pharmacy"  OR  "Clinical Pharmacy Service"  OR  "dispensing"  OR  "drug dispensing"  OR  "health education"  OR  "responsible self medication"  OR  "comprehensive medication management"  OR  "therapeutic drug monitoring"  OR  "drug monitoring"  OR  "medication reconciliation"  OR  "medicines use review"  OR  "brown bag review"  OR  "drug regimen review"  OR  "home medicines review"  OR  "medication therapy review"  OR  "medication management review"  OR  "disease management"  OR  "pharmacotherapeutic follow up"  OR  "medicines management"  OR  "Cognitive service"  OR  "Cognitive services"  OR  "Cognitive pharmaceutical services"  OR  "Cognitive pharmaceutical service"  OR  "Community Pharmacy Services"  OR  "pharmacy services, community"  OR  "Community Pharmacy Service"  OR  "services, community pharmacy"  OR  "Community Pharmaceutic Services"  OR  "Community Pharmaceutical Services"  OR  "Community Pharmaceutical Service"  OR  "service, community pharmacy" ) )  AND  ( TITLE-ABS-KEY ( "Primary Health Care"  OR  "care, primary health"  OR  "health care, primary"  OR  "Primary Healthcare"  OR  "healthcare, primary"  OR  "Primary Care"  OR  "care, primary"  OR  "Basic health care" ) )  AND  ( TITLE-ABS-KEY ( "Pharmacists"  OR  "Pharmacist"  OR  "Clinical Pharmacists"  OR  "Clinical Pharmacist"  OR  "pharmacist, clinical"  OR  "pharmacists, clinical"  OR  "Community Pharmacists"  OR  "Community Pharmacist"  OR  "pharmacist, community"  OR  "pharmacists, community"  OR  "Retail Pharmacists"  OR  "pharmacists, retail"  OR  "Retail Pharmacist"  OR  "pharmacist, retail" ) ) |
| **Lilacs** |
| ((implementation science) OR (implement) OR (implementation) OR (implementation framework) OR (implementation determinants) OR (implementation outcomes) OR (implementation evaluation) OR (active implementation frameworks) OR (adoption) OR (quality improvement) OR (dissemination) OR (complex intervention) OR (implementation research) OR (knowledge translation) OR (knowledge transfer) OR (knowledge mobilization) OR (research utilization) OR (knowledge uptake) OR (knowledge exchange) OR (knowledge translations) OR (translation knowledge) OR (translations knowledge) OR (diffusion innovation) OR (diffusion of innovation) OR (dissemination research) OR (health systems research) OR (operational research) OR (quality improvement research) OR (translational research) OR (knowledge synthesis) ) AND ((pharmaceutical services) OR (services, pharmacy) OR (pharmaceutic services) OR (pharmaceutic service) OR (services, pharmaceutical) OR (pharmaceutical service) OR (service, pharmaceutical) OR (pharmacy services) OR (pharmacy service) OR (service, pharmacy) OR (pharmaceutical care) OR (care, pharmaceutical) OR (management medication therapy) OR (medication therapy management) OR (therapy management medication) OR (drug therapy management) OR (management, drug therapy) OR (medication review) OR (medication reviews) OR (review, medication) OR (reviews, medication) OR (drug utilization review) OR (drug-use review) OR (drug use review) OR (drug-use reviews) OR (reviews, drug-use) OR (review, drug utilization) OR (drug utilization reviews) OR (reviews, drug utilization) OR (utilization review, drug) OR (drug utilization evaluation) OR (drug utilization evaluations) OR (review, drug-use) OR (review, drug use) OR (pharmacy service, clinical) OR (service, clinical pharmacy) OR (clinical pharmacy services) OR (pharmacy services, clinical) OR (services, clinical pharmacy) OR (clinical pharmacy service) OR (dispensing) OR (drug dispensing) OR (health education) OR (responsible self medication) OR (comprehensive medication management) OR (therapeutic drug monitoring) OR (drug monitoring) OR (medication reconciliation) OR (medicines use review) OR (brown bag review) OR (drug regimen review) OR (home medicines review) OR (medication therapy review) OR (medication management review) OR (disease management) OR (pharmacotherapeutic follow up) OR (medicines management) OR (cognitive service) OR (cognitive services) OR (cognitive pharmaceutical services) OR (cognitive pharmaceutical service) OR (community pharmacy services) OR (pharmacy services, community) OR (community pharmacy service) OR (services, community pharmacy) OR (community pharmaceutic services) OR (community pharmaceutical services) OR (community pharmaceutical service) OR (service, community pharmacy)) AND ((pharmacists) OR (pharmacist) OR (clinical pharmacists) OR (clinical pharmacist) OR (pharmacist clinical) OR (pharmacists, clinical) OR (community pharmacists) OR (community pharmacist) OR (pharmacist, community) OR (pharmacists, community) OR (retail pharmacists) OR (pharmacists, retail) OR (retail pharmacist) OR (pharmacist, retail)) AND ((primary health care) OR (care, primary health) OR (health care, primary) OR (primary healthcare) OR (healthcare, primary) OR (primary care) OR (care, primary) OR (basic health care)) AND ( db:("LILACS")) |
| **Web of Science** |
| (((AB=(“Implementation Science” OR “Implement” OR “Implementation” OR “Implementation framework” OR “Implementation determinants” OR “Implementation outcomes” OR “Implementation evaluation” OR “Active Implementation Frameworks” OR “Adoption” OR “Quality improvement” OR “Dissemination” OR “complex intervention” OR “Implementation Research” OR “knowledge translation” OR “knowledge transfer” OR “knowledge mobilization” OR “Research utilization” OR “Knowledge Uptake” OR “knowledge Exchange” OR “Knowledge Translations” OR “translation knowledge” OR “translations knowledge” OR “diffusion innovation” OR “diffusion of innovation” OR “dissemination research” OR “health systems research” OR “operational research” OR “quality improvement research” OR “Translational Research” OR “Knowledge synthesis” )) AND AB=(“Pharmaceutical Services” OR “services, pharmacy” OR “Pharmaceutic Services” OR “Pharmaceutic Service” OR “services, pharmaceutical” OR “Pharmaceutical Service” OR “service, pharmaceutical” OR “Pharmacy Services” OR “Pharmacy Service” OR “service, pharmacy” OR “Pharmaceutical Care” OR “care, pharmaceutical” OR “management medication therapy” OR “Medication Therapy Management” OR “therapy management medication” OR “Drug Therapy Management” OR “management, drug therapy” OR “Medication Review” OR “Medication Reviews” OR “review, medication” OR “reviews, medication” OR “Drug Utilization Review” OR “drug-use review” OR “drug use review” OR “Drug-Use Reviews” OR “reviews, drug-use” OR “review, drug utilization” OR “Drug Utilization Reviews” OR “reviews, drug utilization” OR “utilization review, drug” OR “Drug Utilization Evaluation” OR “Drug Utilization Evaluations” OR “review, drug-use” OR “review, drug use” OR “pharmacy service, clinical” OR “service, clinical pharmacy” OR “Clinical Pharmacy Services” OR “pharmacy services, clinical” OR “services, clinical pharmacy” OR “Clinical Pharmacy Service” OR “dispensing” OR “drug dispensing” OR “health education” OR “responsible self medication” OR “comprehensive medication management” OR “therapeutic drug monitoring” OR “drug monitoring” OR “medication reconciliation” OR “medicines use review” OR “brown bag review” OR “drug regimen review” OR “home medicines review” OR “medication therapy review” OR “medication management review” OR “disease management” OR “pharmacotherapeutic follow up” OR “medicines management” OR “Cognitive service” OR “Cognitive services” OR “Cognitive pharmaceutical services” OR “Cognitive pharmaceutical service” OR “Community Pharmacy Services” OR “pharmacy services, community” OR “Community Pharmacy Service” OR “services, community pharmacy” OR “Community Pharmaceutic Services” OR “Community Pharmaceutical Services” OR “Community Pharmaceutical Service” OR “service, community pharmacy”)) AND AB=(“Primary Health Care” OR “care, primary health” OR “health care, primary” OR “Primary Healthcare” OR “healthcare, primary” OR “Primary Care” OR “care, primary” OR “Basic health care”)) AND AB=(“Pharmacists” OR “Pharmacist” OR “Clinical Pharmacists” OR “Clinical Pharmacist” OR “pharmacist clinical” OR “pharmacists, clinical” OR “Community Pharmacists” OR “Community Pharmacist” OR “pharmacist, community” OR “pharmacists, community” OR “Retail Pharmacists” OR “pharmacists, retail” OR “Retail Pharmacist” OR “pharmacist, retail”) |
| **Central** |
| #1 MeSH descriptor: [Implementation Science] explode all trees  #2 "Implement" OR "Implementation" OR "Implementation framework" OR "Implementation determinants" OR "Implementation outcomes" OR "Implementation evaluation" OR "Active Implementation Frameworks" OR "Adoption" OR "Quality improvement" OR "Dissemination" OR "complex intervention" OR "Implementation Research" OR "knowledge translation" OR "knowledge transfer" OR "knowledge mobilization" OR "Research utilization" OR "Knowledge Uptake" OR "knowledge Exchange" OR "Knowledge Translations" OR "translation knowledge" OR "translations knowledge" OR "diffusion innovation" OR "diffusion of innovation" OR "dissemination research" OR "Implementation Research" OR "health systems research" OR "operational research" OR "quality improvement research" OR "Translational Research" OR "Knowledge synthesis"  #3 #1 OR #2  #4 MeSH descriptor: [Pharmaceutical Services] explode all trees  #5 "services, pharmacy" OR "Pharmaceutic Services" OR "Pharmaceutic Service" OR "services, pharmaceutical" OR "Pharmaceutical Service" OR "service, pharmaceutical" OR "Pharmacy Services" OR "Pharmacy Service" OR "service, pharmacy" OR "Pharmaceutical Care" OR "care, pharmaceutical" OR "management medication therapy"  #6 MeSH descriptor: [Medication Therapy Management] explode all trees  #7 "therapy management medication" OR "Drug Therapy Management" OR "management, drug therapy"  #8 MeSH descriptor: [Medication Review] explode all trees  #9 "Medication Reviews" OR "review, medication" OR "reviews, medication"  #10 MeSH descriptor: [Drug Utilization Review] explode all trees  #11 "drug-use review" OR "drug use review" OR "Drug-Use Reviews" OR "reviews, drug use" OR "review, drug utilization" OR "Drug Utilization Reviews" OR "reviews, drug utilization" OR "utilization review, drug" OR "Drug Utilization Evaluation" OR "Drug Utilization Evaluations" OR "review, drug-use" OR "review, drug use" OR "pharmacy service, clinical" OR "service, clinical pharmacy" OR "Clinical Pharmacy Services" OR "pharmacy services, clinical" OR "services, clinical pharmacy" OR "Clinical Pharmacy Service" OR "dispensing" OR "drug dispensing" OR "health education" OR "responsible self medication" OR "comprehensive medication management" OR "therapeutic drug monitoring" OR "drug monitoring" OR "medication reconciliation" OR "medicines use review" OR "brown bag review" OR "drug regimen review" OR "home medicines review" OR "medication therapy review" OR "medication management review" OR "disease management" OR "pharmacotherapeutic follow up" OR "medicines management" OR "Cognitive service" OR "Cognitive services" OR "Cognitive pharmaceutical services" OR "Cognitive pharmaceutical service"  #12 MeSH descriptor: [Community Pharmacy Services] explode all trees  #13 "pharmacy, services community" OR "Community Pharmacy, Service" OR "services, community pharmacy" OR "Community Pharmaceutic Services" OR "Community Pharmaceutical Services" OR "Community Pharmaceutical Service" OR "service, community pharmacy"  #14 #4 OR #5 OR #6 OR #7 OR #8 OR #9 OR #10 OR #11 OR #12 OR #13  #15 MeSH descriptor: [Primary Health Care] explode all trees  #16 "care, primary health" OR "health, care primary" OR "Primary Healthcare" OR "healthcare, primary" OR "Primary Care" OR "care, primary" OR "Basic health care"  #17 #15 OR #16  #18 MeSH descriptor: [Pharmacists] explode all trees  #19 "Pharmacist" OR "Clinical Pharmacists" OR "Clinical Pharmacist" OR "pharmacist, clinical" OR "pharmacists, clinical" OR "Community Pharmacists" OR "Community Pharmacist" OR "pharmacist, community" OR "pharmacists, community" OR "Retail Pharmacists" OR "pharmacists, retail" OR "Retail Pharmacist", “pharmacist, retail”  #20 #18 OR #19  #21 #3 AND #14 AND #17 AND #20 |
| **The Digital Library of Theses and Dissertations of the University of São Paulo** |
| (“Implementation Science” OR “Implement” OR “Implementation” OR “Implementation framework” OR “Implementation determinants” OR “Implementation outcomes” OR “Implementation evaluation” OR “Active Implementation Frameworks” OR “Adoption” OR “Quality improvement” OR “Dissemination” OR “complex intervention” OR “Implementation Research” OR “knowledge translation” OR “knowledge transfer” OR “knowledge mobilization” OR “Research utilization” OR “Knowledge Uptake” OR “knowledge Exchange” OR “Knowledge Translations” OR “translation knowledge” OR “translations knowledge” OR “diffusion innovation” OR “diffusion of innovation” OR “dissemination research” OR “health systems research” OR “operational research” OR “quality improvement research” OR “Translational Research” OR “Knowledge synthesis”) AND (“Pharmaceutical Services” OR “services, pharmacy” OR “Pharmaceutic Services” OR “Pharmaceutic Service” OR “services, pharmaceutical” OR “Pharmaceutical Service” OR “service, pharmaceutical” OR “Pharmacy Services” OR “Pharmacy Service” OR “service, pharmacy” OR “Pharmaceutical Care” OR “care, pharmaceutical” OR “management medication therapy” OR “Medication Therapy Management” OR “therapy management medication” OR “Drug Therapy Management” OR “management, drug therapy” OR “Medication Review” OR “Medication Reviews” OR “review, medication” OR “reviews, medication” OR “Drug Utilization Review” OR “drug-use review” OR “drug use review” OR “Drug-Use Reviews” OR “reviews, drug-use” OR “review, drug utilization” OR “Drug Utilization Reviews” OR “reviews, drug utilization” OR “utilization review, drug” OR “Drug Utilization Evaluation” OR “Drug Utilization Evaluations” OR “review, drug-use” OR “review, drug use” OR “pharmacy service, clinical” OR “service, clinical pharmacy” OR “Clinical Pharmacy Services” OR “pharmacy services, clinical” OR “services, clinical pharmacy” OR “Clinical Pharmacy Service” OR “dispensing” OR “drug dispensing” OR “health education” OR “responsible self medication” OR “comprehensive medication management” OR “therapeutic drug monitoring” OR “drug monitoring” OR “medication reconciliation” OR “medicines use review” OR “brown bag review” OR “drug regimen review” OR “home medicines review” OR “medication therapy review” OR “medication management review” OR “disease management” OR “pharmacotherapeutic follow up” OR “medicines management” OR “Cognitive service” OR “Cognitive services” OR “Cognitive pharmaceutical services” OR “Cognitive pharmaceutical service” OR “Community Pharmacy Services” OR “pharmacy services, community” OR “Community Pharmacy Service” OR “services, community pharmacy” OR “Community Pharmaceutic Services” OR “Community Pharmaceutical Services” OR “Community Pharmaceutical Service” OR “service, community pharmacy”) AND (“Primary Health Care” OR “care, primary health” OR “health care, primary” OR “Primary Healthcare” OR “healthcare, primary” OR “Primary Care” OR “care, primary” OR “Basic health care”) AND (“Pharmacists” OR “Pharmacist” OR “Clinical Pharmacists” OR “Clinical Pharmacist” OR “pharmacist clinical” OR “pharmacists, clinical” OR “Community Pharmacists” OR “Community Pharmacist” OR “pharmacist, community” OR “pharmacists, community” OR “Retail Pharmacists” OR “pharmacists, retail” OR “Retail Pharmacist” OR “pharmacist, retail”) |
