# Supplementary material for: Implementation of Clinical Pharmacy Services in Primary Health Care: A Scoping Review
Source: J Eval Clin Pract. 2025 Sep 25;31(6):e70285. doi: 10.1111/jep.70285 (PMC12462563; doi:10.1111/jep.70285)
Supplement: Supplementary file 2 — Supplementary Material 3. [file JEP-31-0-s001.docx]

**Supplementary material 3**

**Figure 1.** Flow diagram with the results of the study selection stage. Adapted from Preferred Reporting Items for Systematic Reviews and Meta-Analyses Extension for scoping review Checklist (PRISMA-ScR).


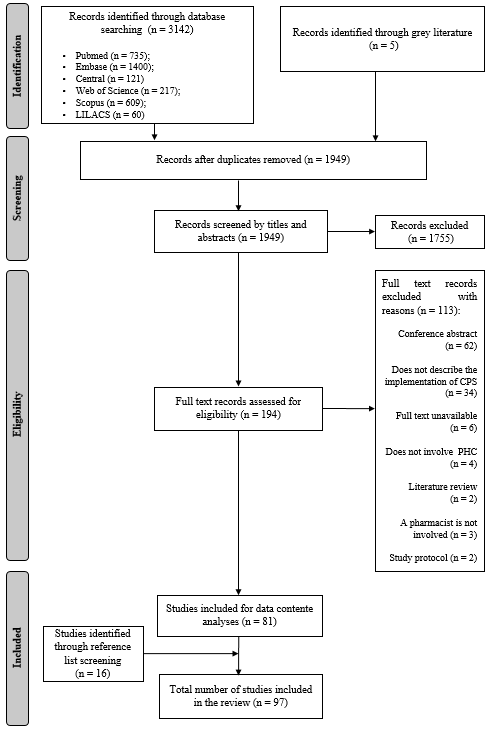


CPS – Clinical Pharmacy Services; PHC – Primary Health Care.
