# Supplementary material for: Implementation of Clinical Pharmacy Services in Primary Health Care: A Scoping Review
Source: J Eval Clin Pract. 2025 Sep 25;31(6):e70285. doi: 10.1111/jep.70285 (PMC12462563; doi:10.1111/jep.70285)
Supplement: Supplementary file 3 — Supplementary Material 4_1. Supplementary Material 4_2. [file JEP-31-0-s003.docx]

**Supplementary Material 4 –** Variables extracted from the studies selected for the scope review

**Table 1.**

Characteristics of studies on the implementation of clinical pharmacy services in primary health care.

| Author (Year) | Country | Objective | Study design | Participants | Type of CPS | Implementation model | Implementation phase* | Location of CPS delivery | | Implementation outcomes |
| --- | --- | --- | --- | --- | --- | --- | --- | --- | --- | --- |
| Xiao, Jie et al. (2023)^59^ | China | To evaluate the effects and influencing factors of physician-pharmacist collaborative clinics in the management of type 2 diabetes mellitus in PHC | Mixed methods study | Patients, pharmacists and doctors | Comprehensive medication management | N/D | Phase 1 | | PHC clinic | Appropriateness |
| Elizabeth J. Austin et al. (2023)^60^ | USA | To describe the implementation barriers and facilitators identified during the formative evaluation that should be leveraged and addressed in future care models [Collaborative Care Model for Pharmacists, Physicians, and Patient Navigators (PPP-CCM)] for the treatment of hepatitis C among patients who inject drugs. | Qualitative study | Physicians, community pharmacists and patient navigators | Drug dispensing and Health education | CFIR | Phase 2 | | PHC clinic | Acceptability and feasibility |

(continued on next page)

Table 1. (continued)

| Author (Year) | Country | Objective | Study design | Participants | Type of CPS | Implementation model | Implementation phase* | Location of CPS delivery | | Implementation outcomes |
| --- | --- | --- | --- | --- | --- | --- | --- | --- | --- | --- |
| Wong YJ, Thum CC, Ng KY, Lee SHW. (2023)^61^ | Malaysia | To assess the feasibility of directly observed treatment for tuberculosis (TB-DOT) in community pharmacies in Malaysia. | Mixed methods study | Community pharmacists | Disease management | CFIR | Pre-implementation (according to the authors) | | Community pharmacy | Appropriateness and feasibility |
| Nabergoj Makovec, Urska et al. (2023)^62^ | Slovenia | To assess the perspective of general practitioners on the implementation of a pharmacist-led medication review service at the Ljubljana Community Health Centre. | Prospective observational study | General practitioners | Medication review | SPO | Phases 2 and 3 | | PHC clinic | Acceptability and feasibility |
| Cerqueira, S.S. et al. (2023)^63^ | Brazil | To understand the factors that may influence the implementation of drug dispensing in community pharmacies, according to pharmacists' perceptions. | Qualitative study | Community pharmacists | Drug dispensing | Problem-based learning using the Maguerez Arch in association with the Apoteca platform | Phase 2 | | Community pharmacy | Feasibility |

(continued on next page)

Table 1. (continued)

| Author (Year) | Country | Objective | Study design | Participants | Type of CPS | Implementation model | Implementation phase* | Location of CPS delivery | | Implementation outcomes |
| --- | --- | --- | --- | --- | --- | --- | --- | --- | --- | --- |
| Hayley C.G. et al. (2022)^64^ | England | To develop and evaluate the feasibility of a mental health support service in community pharmacies, called AMPLIPHY (Advancing Mental Health Provision In Pharmacy). | Mixed methods study | Patients and community pharmacists | Comprehensive medication management | N/D | Phase 1 | | Community pharmacy | Feasibility |
| Erin F. Barreto et al. (2022)^65^ | USA | To describe the development and feasibility of a new multidisciplinary approach for patients with kidney injury during acute care transition (ACT). | N/D | Patients | Drug monitoring, Medication reconciliation, Medication review, and Health education. | N/D | Phases 1 and 3 | | PHC clinic | Appropriateness and feasibility |
| Barros, D. S.L.; da Silva, D. L. M.; Leite, S. N. (2022)^66^ | Brazil | To understand the strengths and weaknesses in the development of clinical services in primary health care in the Federal District. | Qualitative study | Pharmacists at the Basic Health Units (BHUs) | CPS in general | SWOT | Phases 1 and 2 | | BHU | Feasibility |
| Mary, M. et al., (2022)^67^ | England | To identify factors that affect the initial implementation of the Structured Medication Reviews (SMRs). | Qualitative study | Clinical pharmacists | Medication review | N/D | Phases 1 and 2 | | PHC clinics | Feasibility |

(continued on next page)

Table 1. (continued)

| Author (Year) | Country | Objective | Study design | Participants | Type of CPS | Implementation model | Implementation phase* | Location of CPS delivery | | Implementation outcomes |
| --- | --- | --- | --- | --- | --- | --- | --- | --- | --- | --- |
| Taylor, S; Cairns, A; Glass, B. D.  (2022)^68^ | Australia | To evaluate the feasibility, accessibility, and acceptability of a pharmacist-led intervention for ear diseases in rural consumers who attended the community pharmacy. | Mixed methods study before and after intervention | Community pharmacists, general practitioners, patients. | Management of Minor Ilness | PRECEDE and PROCEED | Phase 3 | | Community pharmacy | Acceptability, feasibility and penetration |
| Royals, C. et al. (2022)^69^ | USA | To evaluate the clinical value, economic impact, and patient satisfaction regarding Medicare Annual Wellness Visits (AWVs), newly implemented and pharmacist-led, in a rural Federally Qualified Health Center (FQHC). | Retrospective cohort study | Patients | Medication review | N/D | Phases 1 and 3 | | PHC clinic | Acceptability, feasibility, clinical and humanistic results. |
| Lara-Júnior, C.R. et al. (2022)^70^ | Brazil | To evaluate the implementation and effectiveness of a Comprehensive medication review service for individuals with tuberculosis in the city of Belo Horizonte. | Hybrid effectiveness implementation study | Patients | Comprehensive medication review | N/D | Phase 3 | | BHU | Feasibility and clinical outcomes |

(continued on next page)

Table 1. (continued)

| Author (Year) | Country | Objective | Study design | Participants | Type of CPS | Implementation model | Implementation phase* | Location of CPS delivery | | Implementation outcomes |
| --- | --- | --- | --- | --- | --- | --- | --- | --- | --- | --- |
| En‐Nasery‐de Heer, S. et al. (2022)^71^ | the Netherlands | To evaluate the implementation fidelity of the care transition program  "Medication Actions to Reduce Hospitalizations through a Collaboration of Community and Hospital Pharmacists" (MARCH). | Mixed methods study | Community pharmacists, hospital pharmacists, pharmacy technicians, and consultant pharmacists. | Medication reconciliation and Medication review | CFIF | Phase 3 | | Community pharmacy and households | Penetration and fidelity |
| D’Andréa, R.D.; Wagner, G. A.; Schveitzer, M. C.  (2022)^72^ | Brazil | To describe the perceptions of PHC pharmacists regarding the implementation of pharmaceutical care in a region of the municipality of São Paulo. | Qualitative study | Pharmacists | CPS in general | N/D | Phases 1 and 2 | | BHU | Feasibility |
| Claire, M; Claire, A; Matthew, B.  (2022)^73^ | England | To examine the implementation experience of clinical pharmacists in the role of general practitioners, evaluating the impact, perspectives on the role, and the barriers and facilitators to the implementation process. | Mixed methods study | Patients, pharmacists, general practitioners and clinical practice assistants. | CPS in general | SWOT | Phases 2 and 3 | | PHC clinic | Acceptability, feasibility, penetration, clinical and humanistic results. |

(continued on next page)

Table 1. (continued)

| Author (Year) | Country | Objective | Study design | Participants | Type of CPS | Implementation model | Implementation phase* | Location of CPS delivery | | Implementation outcomes |
| --- | --- | --- | --- | --- | --- | --- | --- | --- | --- | --- |
| Blanchard, C. M. et al. (2021)^74^ | USA | To describe the strategies used to implement the service within a team-based home care program and evaluate the implementation process by measuring outcomes such as adoption, acceptability, appropriateness, feasibility, fidelity, coverage, and sustainability. | Mixed methods study | Clinical pharmacists, managers and other health professionals | Comprehensive medication management | Proctor et al. ERIC | Phases 1, 2 and 3 | | Households | Adoption, acceptability, feasibility, fidelity, penetration, sustainability, and humanistic outcomes. |
| Lee, P. Y. et al., (2021)^75^ | Malaysia | To explore the opinions of healthcare professionals regarding the barriers faced in the implementation of supported self-management of asthma in adults in a primary care setting in Malaysia, considering the country’s cultural and socioeconomic contexts. | Qualitative study | Pharmacists, doctors and nurses | Disease management | N/D | Phase 2 | | PHC clinic | Feasibility |

(continued on next page)

Table 1. (continued)

| Author (Year) | Country | Objective | Study design | Participants | Type of CPS | Implementation model | Implementation phase* | Location of CPS delivery | Implementation outcomes |
| --- | --- | --- | --- | --- | --- | --- | --- | --- | --- |
| Bawab, N. et al. (2021)^76^ | Switzerland | To describe the implementation, from the perspectives of the pharmaceutical team, of a care model for patients with type 2 diabetes mellitus, Siscare-DT2, in the daily practice of primary care in the French-speaking part of Switzerland. | Hybrid effectiveness implementation study | Pharmacists and patients | Disease management | FISpH | Phase 3 | Community pharmacy | Adoption, viability, acceptability, penetration, fidelity, cost-implementation, sustainability, and economic outcomes. |
| Dos Santos Júnior, G.A. et al.  (2021)^77^ | Brazil | To evaluate the implementation of a clinical pharmacy service in several public health units in a major city in northeastern Brazil. | Quasi-experimental before-and-after study. | Pharmacists, local managers, patients, physicians, nurses, social workers, and community health workers. | Comprehensive medication management | Problem-based learning using the Maguerez Arch in association with the APOTECA framework | Phases 1, 2 and 3 | BHU | Appropriateness, feasibility and clinical outcomes |
| Albertson, S et al.  (2021)^78^ | USA | To determine the clinical impact and sustainability of the implementation of clinical pharmacy services in a clinic for homeless adults. | N/D | Patients | Comprehensive medication management | N/D | Phases 1 and 3 | PHC clinics | Appropriateness, cost-implementation and clinical outcomes |

(continued on next page)

Table 1. (continued)

| Author (Year) | Country | Objective | Study design | Participants | Type of CPS | Implementation model | Implementation phase* | Location of CPS delivery | | Implementation outcomes |
| --- | --- | --- | --- | --- | --- | --- | --- | --- | --- | --- |
| Do, V et al.  (2021)^79^ | USA | To describe the implementation of a pharmacist-led care transition model for post-transplant hyperglycemia. | N/D | Patients | Comprehensive medication management | N/D | Phase 3 | | PHC outpatient clinic | Feasibility and clinical outcomes |
| Cheong, A. T. et al.  (2021)^80^ | Malaysia | To determine the clinical impact and sustainability of the implementation of clinical pharmacy services in a clinic for homeless adults. | Qualitative study | Doctors, nurses, pharmacists and health aides | Disease management | N/D | Phase 2 | | PHC clinic | Feasibility |
| García-Agua Soler, N. et al.  (2021)^81^ | Spain | To conduct a study on the initial implementation of the Pharmacotherapy Review service (Revisa project) in Spanish community pharmacies. | Cross-sectional study | Community pharmacists and patients | Medication review | N/D | Phase 3 | | Community pharmacy | Acceptability, feasibility, cost-implementation and clinical, economic and humanistic outcomes. |
| Hashmi, F. et al. (2021)^82^ | Pakistan | To explore the perspectives and readiness of community pharmacists regarding practice changes and the facilitators and obstacles associated with Clinical Pharmacy Services (CPS). | Mixed methods study | Community pharmacists | CPS in general | N/D | Phases 1 and 2 | | Community pharmacy | Feasibility |

(continued on next page)

Table 1. (continued)

| Author (Year) | Country | Objective | Study design | Participants | Type of CPS | Implementation model | Implementation phase* | Location of CPS delivery | | Implementation outcomes |
| --- | --- | --- | --- | --- | --- | --- | --- | --- | --- | --- |
| Qazi, A. et al.  (2021)^83^ | Australia | To gather pharmacists' opinions and recommendations on pragmatic models of collaboration between community pharmacists and general practitioners in the provision of asthma management services in future service delivery models. | Qualitative study | Community pharmacists | Disease management | N/D | Phase 2 | | Community pharmacy | Feasibility |
| Risøy, A. J. et al.  (2021)^84^ | Norway | To explore the experience of Norwegian pharmacists in a diabetes risk assessment service, including analytical quality control, in a community pharmacy setting. | Qualitative study | Community pharmacists | Disease screening | N/D | Phases 2 and 3 | | Community pharmacy | Acceptability and feasibility |
| Turner, K. et al.  (2021)^85^ | USA | To examine the implementation process of community pharmacies in North Carolina participating in a Medicaid population health management intervention. | N/D | Community pharmacy representatives, pharmacists, owners and technicians. | Medication review | Rogers' theory (2010) | Phases 1, 2 and 3 | | Community pharmacy | Adoption, appropriateness and sustainability |

(continued on next page)

Table 1. (continued)

| Author (Year) | Country | Objective | Study design | Participants | Type of CPS | Implementation model | Implementation phase* | Location of CPS delivery | | Implementation outcomes |
| --- | --- | --- | --- | --- | --- | --- | --- | --- | --- | --- |
| Okuyan, B. et al.  (2021)^86^ | Turkey | To determine the barriers and facilitators perceived by community pharmacists during the provision of pharmaceutical care to older patients, using a theoretical domains framework. | Cross-sectional study | Community pharmacists | CPS in general | Theoretical Domains Framework (TDF) | Phase 2 | | Community pharmacy | Feasibility |
| Guilcher, S. J. T. et al. (2020)^87^ | Canada | To evaluate the acceptability, feasibility, fidelity, and factors influencing the implementation process of PROMPT in the transition of care from hospital to community. | Mixed methods studies | Community and hospital pharmacists. | Medication reconciliation | CFIR | Phases 2 and 3 | | Community pharmacy | Acceptability, feasibility, fidelity and humanistic results. |
| Collin et al.,  (2020)^88^ | USA | Pilot implementation study of a pharmacotherapy review service for older patients using potentially inappropriate medications for this population, within a Medicare annual wellness visit program. | N/D | Clinical pharmacist and patients | Medication review and Medication reconciliation | N/D | Phases 1 and 2 | | PHC clinic | Appropriateness and feasibility |

(continued on next page)

Table 1. (continued)

| Author (Year) | Country | Objective | Study design | Participants | Type of CPS | Implementation model | Implementation phase* | Location of CPS delivery | | Implementation outcomes |
| --- | --- | --- | --- | --- | --- | --- | --- | --- | --- | --- |
| Patrick, R. et al.  (2020)^89^ | Ireland | To explore the barriers and facilitators with healthcare professionals for the implementation of medication reconciliation within primary and secondary care in Ireland. | Qualitative study | Community pharmacists, hospital pharmacists, hospital consultants, general practitioners and hospital physicians. | Medication reconciliation | Theoretical framework - adapted from van Sluisveld et al. 2012 | Phases 1 and 2 | | Community pharmacy | Feasibility |
| Turner, K. et al.  (2020)^90^ | USA | To describe the implementation approach of comprehensive medication management in the primary care setting of the University of Utah Health and the evaluation of implementation fidelity. | N/D | Clinical pharmacists | Comprehensive medication management | AIFs | Phase 3 | | PHC clinic | Adoption and fidelity |
| Varas-Doval, R. et al.  (2020)^91^ | Spain | To evaluate an implementation program for a medication review service in community pharmacy, comparing clinical and humanistic outcomes with those from a previously conducted randomized controlled trial. | Hybrid effectiveness implementation study | Community pharmacists and patients. | Medication review | FISpH | Phases 1 and 3 | | Community pharmacy | Penetration, fidelity, sustainability and clinical outcomes. |

(continued on next page)

Table 1. (continued)

| Author (Year) | Country | Objective | Study design | Participants | Type of CPS | Implementation model | Implementation phase* | Location of CPS delivery | | Implementation outcomes |
| --- | --- | --- | --- | --- | --- | --- | --- | --- | --- | --- |
| Mc Namara, K. P. et al.  (2020)^92^ | Australia | To evaluate the impact, acceptability, and feasibility of the Cardiovascular Absolute Risk Screening (CARS) program in screening patients with cardiovascular risk | Mixed methods study | Patients and community pharmacists | Disease screening | N/D | Phases 2 and 3 | | Community pharmacy | Appropriateness, feasibility and clinical outcomes. |
| Rhinehart, H. E. et al.  (2020)^93^ | USA | To evaluate the impact of a pharmacist-led post-surgical discharge medication reconciliation program in a multidisciplinary PHC network in Ohio. | N/D | Patients | Medication reconciliation | N/D | Phase 3 | | PHC outpatient clinic | Feasibility and clinical outcomes |
| Barry, H. E. et al.  (2020)^94^ | Ireland | To develop an intervention to improve medication management for patients with dementia in primary care. | Qualitative study | Community pharmacists and general practitioners | Medication review | Theoretical Domains Framework (TDF) | Phase 1 | | Community pharmacy | Appropriateness |
| Qazi, A; Armour, C; Saini, B. (2020)^95^ | Australia | To gather the opinion of general practitioners regarding the pragmatic collaboration model between community pharmacists and general practitioners for better asthma management. | Qualitative study | General practitioners | Medication review and Health education | N/D | Phase 2 | | Community pharmacy | Feasibility |

(continued on next page)

Table 1. (continued)

| Author (Year) | Country | Objective | Study design | Participants | Type of CPS | Implementation model | Implementation phase* | Location of CPS delivery | | Implementation outcomes |
| --- | --- | --- | --- | --- | --- | --- | --- | --- | --- | --- |
| Hattingh, L. et al.  (2020)^96^ | Australia | To explore the factors that contributed to the successful implementation and sustainability of CPS in Australian community pharmacies. | Mixed methods study | Community pharmacists | CPS in general | COM-B | Phases 2 and 3 | | Community pharmacy | Acceptability and feasibility |
| ALY, M. et al. (2019)^97^ | United Kingdom | To explore the opinions and experiences of community pharmacists, academics, policymakers, and professional organizations involved in the implementation of management of minor ilness in the United Kingdom. | Qualitative study | Community pharmacists, policymaker, pharmacy academics, and professional organization, such as pharmacy technicians, managers (clinical, retail, IT) | Management of minor ilness | N/D | Phases 2 and 3 | | Community pharmacy | Appropriateness and feasibility |
| Mantzouran, E. et al.  (2019)^98^ | Wales | To explore the views and opinions of community pharmacists regarding their initial experience and preparedness levels for the Sore Throat Test and Treat (STTT) service in Wales. | Qualitative study | Community pharmacists | Disease screening and Comprehensive medication management | N/D | Phases 1 and 2 | | Community pharmacy | Acceptability and feasibility |

(continued on next page)

Table 1. (continued)

| Author (Year) | Country | Objective | Study design | Participants | Type of CPS | Implementation model | Implementation phase* | Location of CPS delivery | | Implementation outcomes |
| --- | --- | --- | --- | --- | --- | --- | --- | --- | --- | --- |
| Graham, J. et al. (2019)^99^ | USA | To evaluate community pharmacists' perceptions of the information, communication, and care provided to recently discharged hospital patients, before and after the intervention. | Cross-sectional study | Community pharmacists | Comprehensive medication management | N/D | Phases 1 and 3 | | Community pharmacy | Acceptability |
| Wright, E. A. et al.  (2019)^100^ | USA | To determine the impact of pharmacist-provided care and electronic communication on readmissions in a high-risk patient group. | Pragmatic clinical trial | Patients | Comprehensive medication management | N/D | Phase 3 | | Community pharmacy | Feasibility, cost-implementation, sustainability, clinical and economic outcomes. |
| Stuhec, M.; Gorenc, K.; Zelko, E. (2019)^101^ | Slovenia | To determine whether a clinical pharmacist-led medication review service can improve the quality of medication prescribing in older adults treated with polypharmacy in primary care. | Retrospective cohort study | Patients | Medication review | N/D | Phases 1 and 3 | | PHC clinics | Clinical and economic outcomes |

(continued on next page)

Table 1. (continued)

| Author (Year) | Country | Objective | Study design | Participants | Type of CPS | Implementation model | Implementation phase* | Location of CPS delivery | | Implementation outcomes |
| --- | --- | --- | --- | --- | --- | --- | --- | --- | --- | --- |
| Hall, G. et al.  (2019)^102^ | England | To evaluate the outcomes of the Pharmacy First Extended Care Service, a CPS focused on otorhinolaryngological and ocular conditions in terms of patient satisfaction, adherence, and access to the service. | Mixed methods study | Patients | Management of minor ilness | N/D | Phases 1 and 3 | | Community pharmacy | Clinical and humanistic results |
| Bowers, B. L.; Heffern, C.; Billings, S. (2019)^103^ | USA | To examine the implementation of a pharmacotherapeutic follow-up service by pharmacists integrated into a primary care clinic, evaluating its impact on identifying patients for pharmaceutical services. | N/D | Patients | Comprehensive medication management | N/D | Phase 3 | | PHC clinic | Penetration and feasibility |
| Brajković, A. et al.  (2019)^104^ | Croatia | To describe the implementation of comprehensive medication management and the issues related to the pharmacotherapy of patients with chronic diseases in a health center in Croatia. | Mixed methods study | Patients, general practitioners, managers (in health policies and systems) | Comprehensive medication management | N/D | Pre-implementation and initial implementation (informed by the authors) | | PHC clinics | Appropriateness, feasibility and clinical outcomes |

(continued on next page)

Table 1. (continued)

| Author (Year) | Country | Objective | Study design | Participants | Type of CPS | Implementation model | Implementation phase* | Location of CPS delivery | | Implementation outcomes |
| --- | --- | --- | --- | --- | --- | --- | --- | --- | --- | --- |
| Mes, M. A. et al.  (2019)^105^ | United Kingdom | To explore the perspectives of adults with asthma on the potential of pharmacist-led support for adherence to this condition provided in general practice. | Qualitative study | Patients | Comprehensive medication management | N/D | Phase 2 | | PHC clinic | Feasibility |
| Bates, K. A. et al. (2019)^106^ | USA | To describe the opinion of the healthcare team in the *patient-centered medical home* (PCMH) program regarding the involvement of community pharmacists in patient care and to identify areas where the services provided by pharmacists can enhance the quality of care in their clinics. | Qualitative study | Physicians, nursing assistants and medical assistants | Medication review, Medication reconciliation and Health education | N/D | Phases 2 and 3 | | PHC clinic | Acceptability and feasibility |
| Funk, K. A. et al.  (2019)^107^ | USA | To identify how providers perceive the impact of pharmacotherapeutic follow-up on their professional lives. | N/D | Physicians, nurses and medical assistants | Comprehensive medication management | N/D | Phase 3 | | PHC clinic | Acceptability |

(continued on next page)

Table 1. (continued)

| Author (Year) | Country | Objective | Study design | Participants | Type of CPS | Implementation model | Implementation phase* | Location of CPS delivery | | Implementation outcomes |
| --- | --- | --- | --- | --- | --- | --- | --- | --- | --- | --- |
| Victor, R. G. et al.  (2019)^108^ | USA | To evaluate the sustainability of a CPS for the management of systemic hypertension in non-Hispanic Black individuals in American barbershops. | Randomized controlled trial | Patients | Comprehensive medication management | N/D | Post-implementation (informed by the authors) | | Barbershops | Sustainability and clinical outcomes |
| McMillan, S.S. et al. (2018)^109^ | Australia | To evaluate the effectiveness of a CPS for mental health in community pharmacy. | Before and after intervention study | Patients and community pharmacies | Comprehensive medication management | N/D | Phases 1 and 3 | | Community pharmacy | Adoption, feasibility, clinical and humanistic outcomes |
| Hossain, L. N. et al., (2018)^110^ | Australia | To identify the key practice determinants that influence the implementation of community pharmacy services within a primary care network. | Qualitative study | Pharmacists, patients, general practitioners and health system managers | General community pharmacy services (including CPS) | N/D | Phase 2 | | Community pharmacy | Feasibility |
| Giannitrapani, K. F. et al.  (2018)^111^ | USA | To identify the roles that clinical pharmacists can assume in chronic pain management processes based on primary care teams, and to understand the barriers to assuming these roles. | Qualitative study | Clinical pharmacists, nurses and general internists | Comprehensive medication management | N/D | Phases 2 and 3 | | PHC outpatient clinic | Acceptability and feasibility |

(continued on next page)

Table 1. (continued)

| Author (Year) | Country | Objective | Study design | Participants | Type of CPS | Implementation model | Implementation phase* | Location of CPS delivery | | Implementation outcomes |
| --- | --- | --- | --- | --- | --- | --- | --- | --- | --- | --- |
| Ramos, S. F. et al.  (2018)^112^ | Brazil | To identify the facilitators and strategies involved in the implementation process of clinical pharmacy services in some public health units in a major city in northeastern Brazil. | Qualitative study | Pharmacists and health managers | Comprehensive medication management | N/D | Phases 1 and 2 | | BHU | Feasibility |
| Willeboordse, F. et al. (2018)^113^ | the Netherlands | To obtain information on the fidelity of the implementation of a medication review service (Opti-Med intervention) in older patients with chronic illness. | Mixed methods study | Pharmacists, general practitioners, aged care specialists and patients | Medication review | CFIF | Phase 3 | | PHC clinic | Fidelity |
| Sokol, R. et al.  (2018)^114^ | USA | To describe the implementation of a multidisciplinary team-based approach for the treatment of hepatitis C in various primary care settings. | Case report | N/D | Comprehensive medication management | PDSA | Phase 3 | | PHC clinic | Feasibility and cost-implementation |
| Domiati, S. et al.  (2018)^115^ | Lebanon | To evaluate the knowledge and readiness of community pharmacists for Comprehensive medication management in Lebanon. | Descriptive cross-sectional study | Community pharmacists | Comprehensive medication management | N/D | Phases 1 and 2 | | Community pharmacy | Feasibility |

(continued on next page)

Table 1. (continued)

| Author (Year) | Country | Objective | Study design | Participants | Type of CPS | Implementation model | Implementation phase* | Location of CPS delivery | | Implementation outcomes |
| --- | --- | --- | --- | --- | --- | --- | --- | --- | --- | --- |
| Uhl, M. C. et al.  (2018)^116^ | Germany | To evaluate the barriers and facilitators perceived by patients on polypharmacy for the implementation of a medication review service. | Qualitative study | Patients | Medication review | N/D | Phase 2 | | PHC clinic | Feasibility |
| Dos Santos Júnior, G. A. et al.  (2018)^117^ | Brazil | To identify the perceptions of pharmacists and managers regarding the barriers to the implementation of clinical pharmacy services in some public health units in a major city in northeastern Brazil. | Qualitative study | Pharmacists and health managers | CPS in general | N/D | Phase 2 | | BHU | Feasibility |
| Risøy, A. J. et al.  (2018)^118^ | Norway | To test the feasibility of using a diabetes risk assessment tool followed by HbA1 measurement in a Norwegian community pharmacy setting. | N/D | Community pharmacists and patients | Disease screening | N/D | Phase 3 | | Community pharmacy | Feasibility and clinical outcomes |
| Deal, H. I. et al. (2017)^119^ | Canada | To describe the adoption of the pharmacotherapy review service of the Nova Scotia Seniors Pharmacare (NSSP) program. | Retrospective observational study | Patients and community pharmacies | Medication review | N/D | Phase 3 | | Community pharmacy | Adoption |

(continued on next page)

Table 1. (continued)

| Author (Year) | Country | Objective | Study design | Participants | Type of CPS | Implementation model | Implementation phase* | Location of CPS delivery | | Implementation outcomes |
| --- | --- | --- | --- | --- | --- | --- | --- | --- | --- | --- |
| Hatting, L.H. et al. (2017)^120^ | Australia | To compare community pharmacies that implemented a clinical pharmacy service for mental health with those that did not implement it and to identify the factors involved in the implementation. | Mixed methods study | Community pharmacies and community pharmacists | Comprehensive medication management | N/D | Phases 2 and 3 | | Community pharmacy | Adoption, feasibility, acceptability |
| Gonçalves, M. G. B.  (2017)^121^ | Brazil | To develop a systematic proposal for the pharmacotherapeutic follow-up of patients served in the Primary Health Care and Specialty Networks in the municipality of São Paulo. | Descriptive observational study | Pharmacists | Comprehensive medication management | N/D | Phase 1 | | BHU | Feasibility |
| Willeboordse, F. et al.  (2017)^122^ | the Netherlands | To investigate the effectiveness of medication review on the quality of life of older patients with geriatric issues in general practice. | Randomized controlled trial | Patients | Medication review | N/D | Phase 3 | | PHC clinic | Clinical results |
| Norman, J. L. et al.  (2017)^123^ | USA | To describe the implementation of a pharmacist-managed chronic pain clinic in primary health care. | N/D | Patients, pharmacists and doctors | Comprehensive medication management | N/D | Phase 3 | | PHC outpatient clinic | Acceptability and feasibility |

(continued on next page)

Table 1. (continued)

| Author (Year) | Country | Objective | Study design | Participants | Type of CPS | Implementation model | Implementation phase* | Location of CPS delivery | | Implementation outcomes |
| --- | --- | --- | --- | --- | --- | --- | --- | --- | --- | --- |
| Ensing, H. T. et al.  (2017)^124^ | the Netherlands | To explore the barriers and facilitators influencing the adoption of a post-discharge home visit by community pharmacists. | Mixed methods study | Community pharmacists | Medication review | Conceptual Framework of Greenhalgh et al (2004) | Phase 2 | | Households | Feasibility |
| Sim, T. F. et al.  (2017)^125^ | Australia | To explore the perspectives of community pharmacists on the implementation of breastfeeding-related services, the factors to consider, and the necessary implementation strategies in community pharmacies. | Qualitative study | Community pharmacists | Health education | N/D | Phases 2 and 3 | | Community pharmacy | Acceptability and feasibility |
| Thornley, G. T. et al. (2016)^126^ | England | Pilot study to test the feasibility and benefits of a service implemented in community pharmacies that incorporates rapid antigen detection tests (RADT) for patients aged 12 years and older who present symptoms of sore throat, according to Centor criteria. | N/D | Patients | Disease screening and Comprehensive medication management | N/D | Phases 1 and 3 | | Community pharmacy | Feasibility and clinical outcomes |

(continued on next page)

Table 1. (continued)

| Author (Year) | Country | Objective | Study design | Participants | Type of CPS | Implementation model | Implementation phase* | Location of CPS delivery | | Implementation outcomes |
| --- | --- | --- | --- | --- | --- | --- | --- | --- | --- | --- |
| Gallimore, C. E. et al.  (2016)^127^ | USA | Determine whether the inclusion of a pharmacist conducting medication reviews for patients using psychotropic medications in primary care is feasible and facilitates the safe use of medications. | N/D | Patients and pharmacists | Medication review | N/D | Phases 2 and 3 | | PHC clinic | Acceptability and feasibility |
| Chang, A. R. et al.  (2016)^128^ | USA | Examine the feasibility of comprehensive medication management to improve the screening of proteinuria and the management of chronic kidney disease within a broad and integrated health system. | Pragmatic clinical trial | Patients and clinical pharmacists | Comprehensive medication management | N/D | Phase 3 | | PHC clinic | Acceptability, feasibility and clinical outcomes |
| Perraudin, C.; Fleury, B.; Pelletier-Fleury, N. (2015)^129^ | France | To evaluate the feasibility and effectiveness of a pharmacist-led intervention to improve the recognition of Obstructive Sleep Apnea in primary care. | Cohort study | Patients | Disease screening and Health education | N/D | Phases 1 and 3 | | Community pharmacy | Feasibility and clinical outcomes |

(continued on next page)

Table 1. (continued)

| Author (Year) | Country | Objective | Study design | Participants | Type of CPS | Implementation model | Implementation phase* | Location of CPS delivery | | Implementation outcomes |
| --- | --- | --- | --- | --- | --- | --- | --- | --- | --- | --- |
| Harrison, J.; Shaw, J. P.; Harrison, J. E. (2015)^130^ | New Zealand | To evaluate the quality of anticoagulation control in the Community Pharmacy Anticoagulation Management Service. | Before-and-after intervention study | Patients | Drug monitoring | N/D | Phase 3 | | Community pharmacy | Acceptability, feasibility and clinical outcomes |
| Morrissey, H. et al. (2015)^131^ | Australia | To investigate the sustainability of an intervention proposal for chronic disease management among rural patients in community pharmacy and the barriers to implementation. | N/D | Patients and pharmacists | Medication review and Health education | N/D | Phase 3 | | Rural community pharmacy | Acceptability, feasibility and sustainability |
| Harrison, J.; Shaw, J. P.; Harrison, J. E. (2014)^132^ | New Zealand | To evaluate the attitudes toward a collaborative pharmacy-based care model in patients using warfarin in the community. | Mixed methods study | Pharmacists, general practitioners, nurses and patients | Drug monitoring | N/D | Phases 1 and 3 | | Community pharmacy | Acceptability |
| Pherson, C. E. et al. (2014)^133^ | USA | To explore the viability and impact of a home visit service by pharmacists during care transitions. | N/D | Patients and pharmacists | Medication reconciliation and Health education | N/D | Phases 1 and 3 | | Households | Acceptability and clinical outcomes |

(continued on next page)

Table 1. (continued)

| Author (Year) | Country | Objective | Study design | Participants | Type of CPS | Implementation model | Implementation phase* | Location of CPS delivery | | Implementation outcomes |
| --- | --- | --- | --- | --- | --- | --- | --- | --- | --- | --- |
| Collier, I. A.; Baker, D. M. (2014)^134^ | USA | To describe the implementation of a Veterans Affairs clinic managed by pharmacists for high-risk patients with type 2 diabetes mellitus. | N/D | Patients | Comprehensive medication management | N/A | Phase 3 | | PHC clinic | Sustainability and clinical outcomes |
| Tan, E. C. K. (2014)^135^ | Australia | To obtain the opinions of physicians and pharmacists regarding the integration of pharmacists in general practice in Australia. | Qualitative study | Pharmacists and general practitioners | Medication review | N/D | Phases 1 and 3 | | PHC clinic | Acceptability and feasibility |
| Babiker, A. H.; Clarson, L.; Awaisu, A. (2014)^136^ | Qatar | To evaluate the knowledge, attitudes, and perceptions of community pharmacists regarding the establishment of medication review in patient care. | Descriptive cross-sectional study | Community pharmacists | Medication review | N/D | Phase 2 | | Community pharmacy | Acceptability and feasibility |

(continued on next page)

Table 1. (continued)

| Author (Year) | Country | Objective | Study design | Participants | Type of CPS | Implementation model | Implementation phase* | Location of CPS delivery | | Implementation outcomes |
| --- | --- | --- | --- | --- | --- | --- | --- | --- | --- | --- |
| Brown, S.; Henderson, E.; Sullivan, C. (2014)^137^ | United Kingdom | To evaluate the acceptability of alcohol screening and brief interventions for women accessing emergency hormonal contraception in community pharmacies. | Qualitative study | Patients and community pharmacists | Disease screening and Health education | N/D | Phase 3 | | Community pharmacy | Acceptability and feasibility |
| Larson, S. et al. (2013)^138^ | USA | To evaluate the adoption of the comprehensive medication management program by patients and pharmacists at the Minnesota Department of Human Services. | Non-experimental descriptive study | Community pharmacists | Comprehensive medication management | N/D | Phases 1 and 3 | | Community pharmacy | Adoption |
| Armour, C. L. et al. (2013)^139^ | Australia | To test the viability, effectiveness, and sustainability of a pharmacy service for asthma in primary care. | Pragmatic clinical trial | Community pharmacists and patients | Comprehensive medication management | N/D | Phase 3 | | Community pharmacy | Feasibility, sustainability and clinical outcomes |
| Rosse, S. et al. (2013)^140^ | USA | To develop, implement, and evaluate a depression screening program conducted by pharmacists in a community setting. | Prospective study | Patients | Disease screening | N/D | Phase 3 | | Community pharmacy | Feasibility and clinical outcomes |

(continued on next page)

Table 1. (continued)

| Author (Year) | Country | Objective | Study design | Participants | Type of CPS | Implementation model | Implementation phase* | Location of CPS delivery | | Implementation outcomes |
| --- | --- | --- | --- | --- | --- | --- | --- | --- | --- | --- |
| Costa, J. M.; Pereira, M. L. (2012)^141^ | Brazil | To identify the impressions of the healthcare team regarding the provision of pharmaceutical care services in the primary care unit, approximately one year after the project's completion. | Qualitative study | Physicians and nursing assistants | Comprehensive medication management | N/D | Phase 3 | | BHU | Acceptability |
| Dolor, R. J. et al. (2012)^142^ | USA | To describe the variations and challenges in patient recruitment, enrollment, pharmacist visits, and telephone follow-up in a randomized trial conducted across three health centers. | N/D | Clinical pharmacists and researchers | Comprehensive medication management | N/D | Phase 2 | | PHC outpatient clinic | Feasibility |
| Fuller, L. et al. (2012)^143^ | USA | To implement and evaluate a chronic obstructive pulmonary disease screening service based on spirometry in a chain of community pharmacies. | Prospective study | Patients | Disease screening | N/D | Phase 3 | | Community pharmacy | Feasibility and clinical outcomes |
| Cawley, M. J.; Pacitti, R.; Warning, W. (2011)^144^ | USA | To evaluate the service of a pharmacist-led spirometry clinic to identify abnormalities in respiratory diseases in a primary care medical office. | Retrospective cohort study | Patients | Disease management | N/D | Phases 1 and 3 | | PHC clinic | Appropriateness and clinical outcomes |

(continued on next page)

Table 1. (continued)

| Author (Year) | Country | Objective | Study design | Participants | Type of CPS | Implementation model | Implementation phase* | Location of CPS delivery | | Implementation outcomes |
| --- | --- | --- | --- | --- | --- | --- | --- | --- | --- | --- |
| Stafford, L. et al. (2011)^145^ | Australia | To evaluate the clinical outcomes of a home-based warfarin management program. | Retrospective cohort study | Patients | Comprehensive medication management | N/D | Phases 1 and 3 | | Households | Feasibility and clinical outcomes |
| Winfrey, C. et al. (2011)^146^ | USA | To evaluate the feasibility of implementing a pharmacist-initiated peripheral artery disease screening program in the community setting and to determine the program's capacity to increase the number of identified patients. | Prospective study | Patients | Disease screening | N/D | Phase 3 | | Community pharmacy | Feasibility and clinical outcomes |
| Bryanta, L. J. M. et al. (2010)^147^ | New Zealand | To explore the attitudinal factors that hinder the increased participation of community pharmacists in medication review services conducted in collaboration with general practitioners. | Qualitative study | Community pharmacists | Medication review | N/D | Phase 3 | | Community pharmacy | Acceptability |

(continued on next page)

Table 1. (continued)

| Author (Year) | Country | Objective | Study design | Participants | Type of CPS | Implementation model | Implementation phase* | Location of CPS delivery | | Implementation outcomes |
| --- | --- | --- | --- | --- | --- | --- | --- | --- | --- | --- |
| Bryanta, L. J. M. et al. (2010)^148^ | New Zealand | To determine whether the involvement of community pharmacists in conducting medication reviews, working with general practitioners, improves therapeutic outcomes related to medications for patients. | Randomized controlled trial | Patients | Medication review | N/D | Phases 1 and 3 | | Community pharmacy | Fidelity and clinical outcomes |
| Pereira, M. L. et al. (2009)^149^ | Brazil | To describe the process of philosophical and practical changes involved in the implementation of a pharmaceutical care service in a community pharmacy. | Qualitative study | Community pharmacists | Comprehensive medication management | N/D | Phase 2 | | Community pharmacy | Feasibility |
| Alkhateeb, F. M. et al. (2009)^150^ | USA | To evaluate physicians' attitudes toward pharmacotherapeutic follow-up services provided by pharmacists as part of Medicare Part D. | N/D | General practitioners | Comprehensive medication management | N/D | Phase 3 | | PHC clinic | Acceptability |

(continued on next page)

Table 1. (continued)

| Author (Year) | Country | Objective | Study design | Participants | Type of CPS | Implementation model | Implementation phase* | Location of CPS delivery | | Implementation outcomes |
| --- | --- | --- | --- | --- | --- | --- | --- | --- | --- | --- |
| Uema, S.A. et al. (2008)^151^ | Argentina | To identify the perceived barriers to implementing pharmaceutical care. | Descriptive cross-sectional study | Hospital pharmacists, community pharmacists and PHC clinic pharmacists | CPS in general | N/D | Phase 1 | | N/A | Feasibility |
| Bradley, F. et al (2008)^152^ | England | To explore and identify the key determinants that influence the adoption of the pharmacotherapy review service. | Mixed methods study | Managers, community pharmacists and representatives of the local pharmaceutical committee | Medication review | N/D | Phase 3 | | Community pharmacy | Adoption |
| Reid, F.; Murray, P.; Storrie, M. (2005)^153^ | Scotland | To implement a Hypertension Treatment Clinic led by pharmacists in a general medical clinic. To evaluate the impact of the clinic on blood pressure (BP) control and the prevention of coronary artery disease | N/D | Patients | Comprehensive medication management | N/D | Phase 3 | | PHC clinic | Acceptability, clinical and humanistic results |
| Sorensen, L. et al. (2004)^154^ | Australia | To evaluate the effectiveness of a multidisciplinary model that offers medication review for patients at risk of medication-related accidents in the community. | Pragmatic clinical trial | Community pharmacists, physicians and patients | Medication review | N/D | Phase 3 | | Households | Acceptability, feasibility, cost-implementation, clinical and economic outcomes |

(continued on next page)

Table 1. (continued)

| Author (Year) | Country | Objective | Study design | Participants | Type of CPS | Implementation model | Implementation phase* | Location of CPS delivery | | Implementation outcomes |
| --- | --- | --- | --- | --- | --- | --- | --- | --- | --- | --- |
| Yanchick, J. K. (2000)^155^ | USA | To describe the development and implementation of a comprehensive medication management in the primary care outpatient clinics of a military hospital in the USA. | N/D | Patients | Comprehensive medication management | N/D | Phase 3 | | PHC clinic | Feasibility, cost-effectiveness, clinical and economic outcomes. |

Phase 1 – Pre-implementation planning; Phase 2 – Designing the implementation strategy; Phase 3 – Evaluating the implementation strategy^56^.

AIFs: Active Implementation Framework; APOTECA: Attitudinal, POlitical, TEChnical and Administrative Framework; PHC: Primary Health Care; CFIF: Conceptual Framework for Implementation Fidelity CFIR: Consolidated Framework for Implementation Research; COM-B: Capability, Opportunity, Motivation and Behaviour model; ERIC: Expert Recommendations for Implementing Change; FISpH: Framework for the Implementation of Services in Pharmacy; N/A: not applicable; N/D: not described; PDSA: Plan-Do-Study-Act cycles PRECEDE: Predisposing, Reinforcing, and Enabling Constructs in Educational Diagnosis and Evaluation; PROCEED: Policy, Regulatory, and Organisational Constructs in Educational and Environmental Development; CPS: Clinical Pharmacy Services; SPO: Structure-process-outcomes; SWOT: Strenghts, Weaknesses, Opportunities and Threats; BHU: Basic Health Units;
