# Supplementary material for: Implementation of Clinical Pharmacy Services in Primary Health Care: A Scoping Review
Source: J Eval Clin Pract. 2025 Sep 25;31(6):e70285. doi: 10.1111/jep.70285 (PMC12462563; doi:10.1111/jep.70285)
Supplement: Supplementary file 4 — Supplementary Material 5. [file JEP-31-0-s004.pdf]

“What has been published about the **implementation of clinical pharmacy services** in the context of primary health care?”

Total of 97 studies included in this review

CLINICAL PHARMACY SERVICES IN PRIMARY HEALTH CARE

TYPE OF CPS[1]

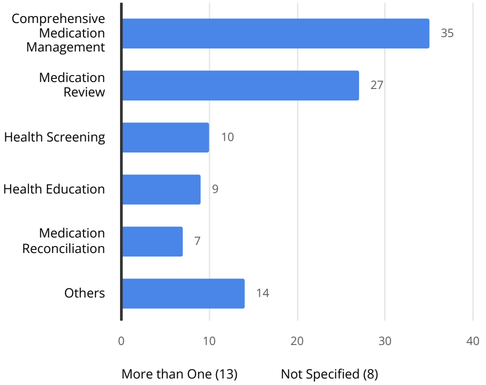

IMPLEMENTATION OUTCOME[2]

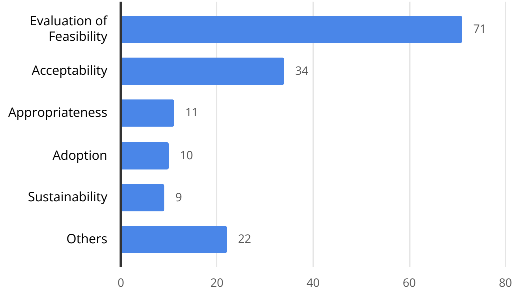

LOCATION OF CPS DELIVERY

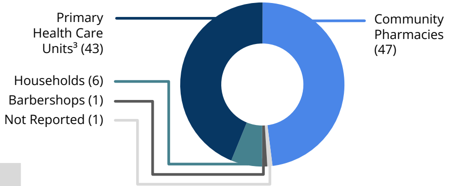

IMPLEMENTATION MODEL

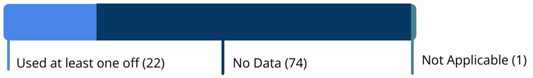

- AIFs
- CFIF
- CFIR
- COM-B
- Conceptual Framework of Greenhalgh et al. (2004)
- ERIC
- FISpH
- PDSA
- PRECEDE and PROCEED
- Problem-based learning using the Maguerez Arch in association with the APOTECA framework
- Problem-based learning using the Maguerez Arch in association with the APOTECA platform
- Proctor et al. (2011)
- Rogers' theory (2010)
- SPO
- SWOT
- Theoretical Domains Framework (TDF)
- Theoretical framework - adapted from van Sluisveld et al. (2012)

IMPLEMENTATION PHASE

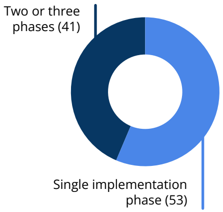

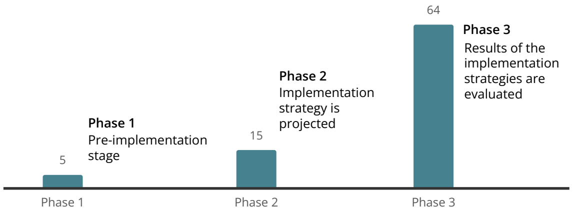

1. CPS (Clinical Pharmacy Services). 2. Proposed by Proctor et al. (2011). 3. Clinics, offices, primary care outpatient facilities, as well as basic health units.
